# Climate Change Impacts on Microbiota in Beach Sand and Water: Looking Ahead

**DOI:** 10.3390/ijerph19031444

**Published:** 2022-01-27

**Authors:** João Brandão, Chelsea Weiskerger, Elisabete Valério, Tarja Pitkänen, Päivi Meriläinen, Lindsay Avolio, Christopher D. Heaney, Michael J. Sadowsky

**Affiliations:** 1 Department of Environmental Health, National Institute of Health Doutor Ricardo Jorge, 1649-016 Lisboa, Portugal; elisabete.valerio@insa.min-saude.pt; 2 Centre for Environmental and Marine Studies (CESAM), Department of Animal Biology, Faculty of Sciences, University of Lisboa, 1749-016 Lisboa, Portugal; 3 Department of Civil and Environmental Engineering, Michigan State University, East Lansing, MI 48824, USA; weiskerg@msu.edu; 4 Department of Health Security, The Finnish Institute for Health and Welfare, 70210 Kuopio, Finland; tarja.pitkanen@thl.fi (T.P.); paivi.merilainen@thl.fi (P.M.); 5 Department of Food Hygiene and Environmental Health, Faculty of Veterinary Medicine, University of Helsinki, 00100 Helsinki, Finland; 6 Department of Environmental Health and Engineering, Johns Hopkins University, Baltimore, MD 21205, USA; lavolio1@jhu.edu (L.A.); cheaney1@jhu.edu (C.D.H.); 7 BioTechnology Institute, University of Minnesota, St. Paul, MN 55108, USA; sadowsky@umn.edu; 8 Department of Soil, Water & Climate, University of Minnesota, St. Paul, MN 55108, USA; 9 Department of Plant and Microbial Biology, University of Minnesota, St. Paul, MN 55108, USA

**Keywords:** climate change, global warming, beach sand, FIB, sand, recreational water, bathing water

## Abstract

Beach sand and water have both shown relevance for human health and their microbiology have been the subjects of study for decades. Recently, the World Health Organization recommended that recreational beach sands be added to the matrices monitored for enterococci and Fungi. Global climate change is affecting beach microbial contamination, via changes to conditions like water temperature, sea level, precipitation, and waves. In addition, the world is changing, and humans travel and relocate, often carrying endemic allochthonous microbiota. Coastal areas are amongst the most frequent relocation choices, especially in regions where desertification is taking place. A warmer future will likely require looking beyond the use of traditional water quality indicators to protect human health, in order to guarantee that waterways are safe to use for bathing and recreation. Finally, since sand is a complex matrix, an alternative set of microbial standards is necessary to guarantee that the health of beach users is protected from both sand and water contaminants. We need to plan for the future safer use of beaches by adapting regulations to a climate-changing world.

## 1. Introduction and Broad Climate Change Projections

**Figure 1:**
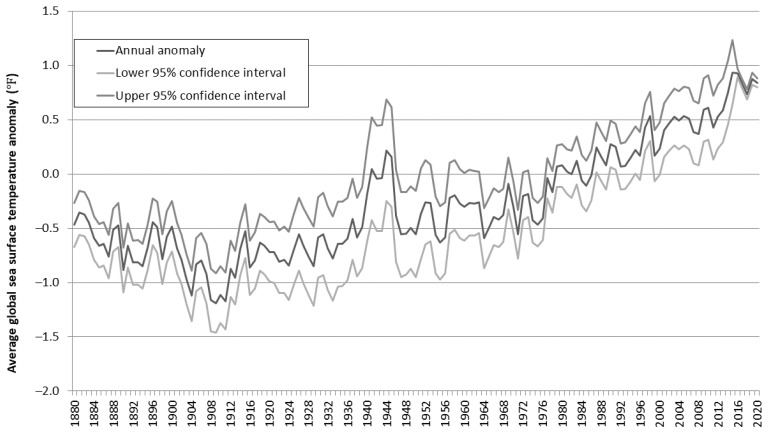
Annual anomaly in average global sea surface temperature in 1880–2020 (adapted from [3]).

Global surface temperatures are predicted to increase by 1–4 °C by the end of this century (2081–2100) relative to those observed from 1986–2005 [1]. The four climate change scenarios presented in the Intergovernmental Panel on Climate Change (IPCC) Synthesis Reports (RCP2.6, RCP4.5, RCP6.0, and RCP8.5) describe the range of predicted changes in climate from mild (RCP2.6) to extreme (RCP8.5), in response to increasing greenhouse gas emissions. It is important to note that, while showing global trends, the predicted changes are not uniform across all regions [2]. Together with a predicted rise in atmospheric temperature, the surface temperature of both fresh and saltwater is also projected to rise. The average global sea surface temperature (SST) has increased steadily for the last 30 years (Figure 1) [3], and the increase in SST is projected to continue, although at a rate slower than the projected rise in the atmospheric temperature. Depending on the emissions scenarios examined, the global SST rise estimates vary from about 1 °C (RCP2.6) to more than 3 °C (RCP8.5). Moreover, the rise in SST is predicted to be highest in subtropical and tropical regions [2].

In addition to temperatures, the global mean sea level is also predicted to increase. Similar to atmospheric and water temperature projections, the predicted sea-level rise depends on the emissions scenario utilized (Table 1). For scenario RCP8.5, the sea level rise during 2081–2100 is predicted to occur at a rate of 8 to 16 mm/year. However, in some regions such as the northern Baltic Sea coastline, the isostatic land uplift is still ongoing after the last ice age and the race between land uplift and sea-level rise will determine the direction in which the coastline will move [4].

Sea level rise has also other implications for recreational beach use. For example, dunes and beach sands are dynamic systems and seasonal tides claim and re-deposit sand from the shores. In some instances, water-driven scour and beach sand removal outweighs re-deposition processes, leading to retracting shorelines.

Current global trends of increasing SST and lake surface temperatures [5,6,7], along with increasing wind speeds and wave heights in coastal regions [8,9], might play a significant role in structuring coastal communities such as kelp forests and shellfish beds (e.g., [10]). Moreover, several predicted climate change scenarios suggest the potential for an increase in human exposure to microbial agents from recreational activities throughout the beach environment, including beach sands and water. The fate and transport of microbes in beach sand are driven by physical, chemical, and biological factors [11]. Weiskerger et al. [12] discussed these mechanisms and dynamics in a previous review. Here, the authors aim to expand upon some of the specific climate change implications for microbiota at recreational beaches, and offer perspectives regarding the future of beach safety in the face of climate change. The discussion herein focuses on providing additional specific details of climate change and how they are projected to impact microbial fate and transport at beaches, supplementing the more holistic and big-picture view presented in Weiskerger et al. [12]. Further, this work aims to expand the analysis to include additional details on the supratidal area of the beach and climate change impacts to fungi, an emerging recreational contaminant and important microbial group affecting public health at recreational sites worldwide [13,14,15,16].

### 1.1. Characterizing Climate Change Impacts on Microbiota

Climate-mediated changes might affect processes controlling inland, estuarine, and coastal concentrations of faecal indicator bacteria (FIB), primarily *Escherichia coli* (*E. coli*), enterococci, pathogens, and the frequency and duration of cyanobacterial blooms, including changes in pH, dissolved oxygen (DO) concentration, salinity (S), dissolved organic carbon (DOC), and dissolved organic nitrogen (DON). Nearshore changes in pH and DO have been documented along the west coast of North America due to several chemical contributions, as well as changes in the intensity of upwelling [17]. These types of changes can be attributable to changes in water temperature and subsequent alterations to water density and potential stratification regimes. Additional changes in pH and DO may also be associated with eutrophication, as has been observed in the Gulf of Mexico and the South China Sea [18]. These two may affect the expected behaviour of FIB in coastal waters and their interactions with sediments, which may be controlled in part by pH. These pH alterations, increases in DOC and DON, and also the increase in water temperature can lead to microbial growth, and changes to some phytoplankton communities (cyanobacteria, diatoms, dinoflagellates, green algae, and chalk-coated *Coccolithophores*) [19]. Subsequently, these changing communities may result in changes to light penetration in the water column, affecting solar-mediated inactivation of indicator organisms and pathogens [20], or perhaps their growth.

The case of cyanobacteria and dinoflagellates is of particular concern since these phytoplanktonic groups are able to develop Harmful Algal Blooms (HABs), as some of their species are able to produce diverse types of toxins, thus causing adverse effects [19,21,22]. These toxins may have a nefarious effect on water associated activities and/or ecology. They can also accumulate in the sand and persist for some time [23], jeopardizing, for instance, recreational activities taking place at those beach environments.

Consequently, both the presence and persistence of waterborne pathogens and indicator microorganisms will likely change in response to climate change. This could have an impact on the concentration of microbiota in near- and foreshore sands. Deposition and scouring processes associated with waves in the nearshore environment [12,24,25] may intensify due to changes in wave dynamics associated with climate change [8,9].

Although not a comprehensive list, Table 2 follows Weiskerger et al. [12] and highlights hypothesized impacts of climate change scenarios on pathogenic microbes in beach sands and adjacent recreational waters. These include: (a) increased temperatures in both fresh and salt waters; (b) altered patterns of precipitation resulting in either flooding or drought; (c) increased wave activity due to rising ocean levels; and (d) an increase in extreme weather events.

Both empirical data collection and mechanistic modelling of FIB are useful tools to help understand the relative importance of the various faecal sources, fate processes, and transport pathways, and for making predictions concerning possible climate change generated phenomena. However, it is important to note that while FIB have been useful to monitor faecal contamination of waterways, there may be differential responses of different microbiota to climate-induced environmental changes, especially once we look into the backshore. FIB may in fact persist or even multiply in the water–sand interface for a longer time than some faecal pathogens [69]. In particular, FIB are unable to predict the growth potential of opportunistic pathogens such as those *Vibrio* species thriving in the water environments [70,71]. Accordingly, models also need to incorporate data from specific pathogens likely to cause human illnesses. For example, Topić et al. [72] describe an outbreak of impetigo in bathers in 2015, linked to the Croatian Vodice beach, due to *Staphylococcus aureus* in water, which was not accompanied by an increase in the levels of FIB. In this paper, 258 locations in Primorje-Gorski Kotar County were investigated for the presence of *S. aureus* in water, as a follow-up of the outbreak, and the results found were that the bacterium was present in between 2.2 and 36.3% of the 2867 samples collected between mid-May and the end of September. The presence of the bacterium correlates with the intensity of human use of the beach. Croatia has very few beaches with sand, but should that be the case, sand could have added to the intensity of the outbreak. Nonetheless, given incomplete information and knowledge of nearshore systems, both empirical and mechanistic models and analyses are needed to illustrate climate change impacts and inform management decisions in the context of environmental and public health in nearshore zones.

### 1.2. Temperature Increases

Rising temperature is a direct response to anthropogenic activities leading to climate change. Moreover, the temperature has a direct effect on the survival and persistence of specific microbial species in both water and sand. Water temperature scenarios are tightly coupled to nearshore hydrodynamics such as stratification, thermal bars, and density-driven currents. An average rise in global atmospheric temperature will result in increases in the temperature of both fresh and marine surface waters, which can directly affect the survival kinetics and growth rates of microbes in both nearshore and upstream tributary environments [73]. However, while it is generally thought that increases in temperature enhance microbial growth, many environmental microbes prefer mesophilic growth temperatures, and elevated temperatures may inhibit the growth of many species. Conversely, rising temperatures may also increase the occurrence of other pathogenic microbes which are capable of multiplying in water environments near body temperature [5,6,7,31,32]. This is especially relevant for fungi when considering the possible selective effect of heatwaves on their potential to infect warm-blooded animals, as described by Casadevall and Kontoyiannis [13] on the emerging *Candida auris*, a multi-resistant yeast of currently high nosocomial impact in multiple regions and whose original habitat is thought to be aquatic [14]. Parasitic protozoa may also benefit from rising temperatures, as described in the new WHO guidelines, namely *Acanthamoeba* and *Naegleria fowleri* [74]. Conversely, the abundance of faecal microbes would decrease in response to many climate change models since faecal microbes generally degrade faster when temperatures increase (i.e., bacterial die-off processes are enhanced) [33,34,35]. For example, Viau et al. [36] and Hokajärvi et al. [37] reported a negative association of the faecal pathogen *Campylobacter* with temperature. Rising temperatures may also increase the occurrence of other pathogenic microbes, which are capable of multiplying in water environments, including in sand, potentially changing the local microbial community [5,6,7,31,32].

Changes in temperature impact physical and biological properties of water in many ways. For example, changing temperatures affect water and contaminant plume buoyancy and dynamics [46]. Changes to relative densities of water and contamination plumes, due to temperature changes in the water column, may lead to increases in surface FIB plumes that are susceptible to solar inactivation. Conversely, changes in relative density may lead to increases in sinking or settling plumes that are more resistant to solar inactivation, yielding potentially higher survival and persistence of contaminants in the environment.

The potential for increased microbial proliferation, in combination with the expansion of the geographic range and seasonality of various tropical pathogens, could pose a significant risk of increases in human exposure. For example, the presence of *Salmonella* spp. in Hawaiian coastal streams has a positive correlation with water temperature [36]. Likewise, the presence and persistence of *Vibrio* spp. is closely and positively correlated with water temperatures [38,39,40]. It has been further suggested that increasing temperatures would allow for increased range and extended seasonality of *Leptospira* spp. [41,42,43]. The effects of increased temperatures also have the potential to alter the persistence of pathogens in beach sand, including methicillin-resistant *Staphylococcus aureus* (MRSA) [44], allergenic fungi, and antifungal resistant fungal species [16].

Another concern is associated with toxic algal blooms, which are also predicted to increase over the next century with the rise of SST and global average lake surface temperature [31,32]. These blooms, along with any microbes that they may be carrying, may be deposited at the sand–water continuum during high tides and high waves, providing an additional input of contaminants to this continuum [45].

In addition to direct changes in the microbial dynamics in the beach ecosystem, temperature rise may have significant indirect effects on environmental conditions and associated microbial loads at beaches. FIB contamination inputs in coastal areas are predicted to change significantly and subsequently may increase adverse health effects for beach users as a result of climate change [75]. Changes in human behaviour may thus challenge beach management paradigms. Examples have been reported in shallow freshwater lakes, where norovirus outbreaks have been associated with a sudden increase in the number of beach users [26]. High temperatures lead to increased beach use, for heat relief, especially if exceptional or extreme weather events occur [27,28]. Such contamination events might have a lesser role in riverine and marine conditions with water flow and larger water volume, respectively. There may also be expanded recreational usage as a result of a growing interest in water sports, ranging from triathlon swims to surfing and wind sailing. Expansion of such recreational use may be particularly prominent at those beaches in proximity to large urban centres. Consequently, direct human inputs may temporarily increase via shedding, leading to increased pathogen loading in beach environments [29,30], or even outbreaks of gastrointestinal illnesses. High temperatures may also attract people to congregate at nearby shores, often at locations that do not comply with recreational water safety standards.

Increased urbanization and climate change may also alter migratory bird patterns, impacting the numbers, species, and behaviour of birds associated with the beach environment. Populations of adaptable bird species, like gulls and Canada geese, have been growing in many urban settings around the world [76], and particularly around the Great Lakes [77]. Urbanisation has been reported as a factor for the presence of pigeon-borne *Cryptococcus* spp. both in sand and in water, which are yeasts responsible for fungal meningitis [16].

Resident populations of birds, particularly shorebirds, can be expected to continue their role as sources of direct animal faecal deposition in the sand. In fact, their removal leads to a dramatic improvement in water quality, as has been reported for example for gulls [78]. Bird population increase in beach settings is expected to lead to a potential increase in human exposure risk to pathogens such as *Salmonella*, *Campylobacter* and *Chlamydia* if populations continue growing in response to climate change [79]. These pathogens, in addition to other zoonotic disease-associated microbiota such as West Nile Virus, *Aspergillus*, *Staphylococcus,* and a variety of antimicrobial-resistant bacteria have been documented in gulls, terns, and barnacle geese [80,81,82]. These microbiota may be transmitted directly via deposition from the birds to coastal environments (sand and water), where they may accumulate and present human health risks. These risks may further be amplified as human beach usage may increase in response to climate change and increasing air temperatures.

### 1.3. Precipitation Increases

Altered precipitation patterns can also greatly influence pathogen exposures at the beach. The intensification of storms, extreme precipitation, and other severe weather events like drought, flooding, storm surge, or even damaging cyclones [1] could cause nearshore inundation, coastal erosion and run-off, introducing pathogenic microbes into coastal waters and on beaches [47,48,49,50,51,52,53,54,57,83,84,85]. Heavy precipitation events, predicted to increase in both frequency and intensity, can cause the resuspension of FIB from beach sand into the water column as well as increases in the dissolved organic matter that can influence solar/UV inactivation of microbial contaminants. These impacts may have subsequent effects on water quality lasting up to 5–7 days after the precipitation event [20,49,57]. Changes in global patterns of precipitation are also predicted to amplify existing direct and anthropogenic impacts of microbiota in coastal environments [11,30]. Increased frequency and intensity of precipitation events can lead to the breakdown of already taxed wastewater infrastructure, resulting in increased point sources of faecal contamination of beaches, such as Combined Sewer Overflow (CSO) events or wastewater effluent [50,58]. Stormwater outfalls frequently present the greatest direct source of faecal contamination, including pathogens, for adjacent surface waters, even in areas with mixed land use [86,87,88]. Further, runoff from impervious surfaces (such as parking lots), CSO discharge events, and stormwater outfall discharges located at or in proximity to beaches can directly contribute to faecal contamination of beaches [55,56].

On the other hand, areas experiencing increased drought may observe increases in microbial exposure in depleted waters, resulting from a higher accumulated concentration and diversity of microbial communities, including pathogens in depleted water supplies [59,60]. Drought conditions may also improve habitat for specific pathogens, including the xerotolerant opportunistic fungal group *Candida*, which can preferentially survive in dry sands [15,61]. Climate change is likely to reduce snowfall amounts and change the timing of snowmelt events, shifting them to earlier in the season, ultimately decreasing snowmelt in the spring, and perpetuating drought conditions in some areas [89].

### 1.4. Wave Activity and Sea Level Rise

Temperature and precipitation changes are two environmental conditions that have been frequently considered as hallmarks of climate change in the literature. Wave activity is not as commonly explored [90] and the effects of waves on the transfer and accumulation of human pathogens in the sand, and their subsequent transfer to recreational water, needs to be studied. We include wave activity here because the sand–water continuum involves dynamic exchanges of microbes between sand and water [12]. Potential climate-induced changes to microbial interactions between water and sand could have important implications on altered exposure conditions [91]. Direct faecal deposition from birds or dogs, or direct runoff onto sand may be distributed over a greater area of beach with increased human activity and extreme weather events, including increased wave activity [54,66,67,92,93,94]. Similarly, periodic tidal rewetting enables FIB and pathogens deposited into dry sands to persist for longer periods [54,68]. Moreover, if ocean depth rises, the tides will edge further inland, allowing for the persistence and exchange of FIB at the sand–water continuum closer to densely populated areas.

Changes in the coastline could also have significant negative impacts on human exposure to pathogens as there has been a global shift towards urbanization with a tendency for cities to develop along coastlines. It has been estimated that nearly half of the world’s population lives within a few hundred kilometres of a coast [95]. The combination of rising sea levels, increasing rainfall amounts and intensity, and increasing urbanization means that the risk of exposure to microbial contamination along the coast will continue to rise. An increase in sea level can also lead to the development of new microbial communities in the sand–water continuum of more inland areas [62,63]. This may precipitate exposure to more diverse microbial contaminants at beaches.

Further, climate changes could result in an increase in gently-sloping coastal areas that could be classified as low-energy for wave action due to sea-level rise. For example, Florida is characterized by flat terrain, which extends outward into the oceans. This platform is composed of a limestone aquifer, which is very shallow in coastal areas. Many beaches and offshore regions are often considered calm and of relatively low energy. As sea levels rise and the coast migrates landward, the topography of terrain similar to south Florida’s could potentially create more areas of low energy, assuming that the platform maintains its integrity as sea level rises [64]. Because of this flat terrain, flooding events will be followed by stranded water, creating low-energy surface water bodies. The increase in flood-prone areas, along with disruptions or overflow of sanitary services, could potentially create pockets of contaminated waters that would not have the benefit of dilution, as is the case for beaches directly connected to the ocean. This, coupled with biofilm dynamics and the influence of low energy waves, could result in changes to how FIB and pathogens are retained along the coast in response to sea-level rise [65].

### 1.5. Economic Impact

There are both direct and indirect costs associated with climate change-induced increases in the number of harmful bacteria and fungi and resulting exposures to humans. While some are obvious, such as loss of wages due to illness, others are more intangible but no less severe. For example, current costs of recreational waterborne illnesses in the US alone approaches $3 billion annually (range of $2.2–$3.7 billion), with moderate to severe illnesses accounting for ~73% of the economic burden [96]. These costs will likely drastically increase due to climate change-induced rises in the number of pathogenic bacteria and fungi present in recreational waters. Moreover, climate-induced increases in temperature, precipitation, sea level, and storm intensities worldwide will likely lead to more disasters and increases in human exposure to pathogens in waterways [12], increases in contact of humans and animals, as well as a greater prevalence of antibiotic and heavy metal resistant bacteria and fungi, primarily due to the greater runoff [97]. These costs are in addition to those required to maintain and enhance infrastructure in a changing environment [98,99].

## 2. Recommendations for the Future

The popularity of human recreational beach activities, combined with predicted climate change scenarios, could amplify the risk of human exposure to pathogens and increase the incidence of illnesses. It has been estimated that 4 billion surface water recreation events occur annually in the US alone [96,100], and the use of water for recreational activities is growing worldwide [101]. If climate variability increases the microbial burden of beach environments, it is likely to result in increased human exposure and resultant disease through recreational contact with water via swimming, watersports, activities at the sand–water continuum [102], and contact with beach sand (expert review by Solo-Gabriele et al. [84]). In addition, human behaviours at the beach can influence the degree and severity of outcomes associated with exposure, especially among sensitive sub-populations (i.e., immune-compromised patients, elderly people, or children) who experience enhanced susceptibility to adverse health effects [11,103]. In fact, evidence suggests that beach sand can harbour higher levels of FIB than the adjacent water, and that sand contact can elevate the risk of human disease outcomes [104,105,106,107,108,109]. As hand-to-mouth behaviours are typical in beachgoers who often eat, drink, and play in the sand or at the sand–water continuum, it is crucial to consider microbial exposures among sensitive sub-groups as well as the general population, within the context of climate-induced pathogen variability predictions and mitigation actions now and into the future. Moreover, disease occurrence is not limited to direct sand and water contact, since strong winds and water activities can generate and propagate aerosolized particles carrying microbiota from what is in the water surface [110] and in the sand [74]. These particles may be inhaled but can also deposited on any exposed skin and mucosal surface or penetrate nostrils and ears, which is particularly relevant for some bacterial and fungal pathogens such as *Mucoraceae* [111] and *Aspergillus* section *Nigri* [112], respectively. 

The traditional microbiological parameters used to assess water and sand quality, are unable to indicate the biological origin of a faecal contamination source. Microbial Source Tracking (MST) is a method that has been used and improved during the last two decades [113], which allows the establishment of an association between some faecal microorganisms with a particular animal host [114]. The association between the faecal contamination detected and the host enables the management authorities to take the most suitable measures to mitigate the problem and thus allow the safeguarding of public health. This methodology was used, for example, in an episode where sand contamination was detected and several biological sources (including canine) were identified [115].

Quantitative Microbial Risk Assessment (QMRA) [116,117] is a widely used tool for determining the health effects of waterborne pathogens. It has been used, to assess the extent of microbial contamination in food [118] and water [119,120,121], as well as in beach environments for calculating infection risks to recreational water users [122,123,124,125,126]. This approach takes into consideration microbial exposure levels, which might change in response to a changing climate [48,120,127]. QMRA has helped advance the field beyond FIB to an informed understanding of the contribution of specific pathogens to enteric diseases associated with recreational beach activities, and to fill knowledge gaps that remain from studying FIB levels alone [122,128,129,130,131]. This framework can assess the public health impacts of a changing microbiome at beaches and may enable more accurate management actions to prevent potential climate change-driven health impacts. With the emergence of new contaminants of human health concern, as well as projected effects of climate change on microbial communities in beach systems, the development and use of QMRA will depend on additional epidemiological and exposure pathway data collection. Therefore, it is important that future research in this area account for these emerging and changing conditions in order to effectively model and manage health risks at beaches.

While a statistical relationship between waterborne diarrheal diseases and severe weather-related events has been observed in several studies [132,133,134,135,136], the literature lacks a comprehensive investigation of long-term microbial exposure trends associated with climate, especially in the context of beach sand-related microbial exposure. While there have been some longer-term change detection studies that can aid in the understanding of climate change impacts on the environment, these can be time and cost-intensive and are therefore rare [137,138,139,140]. Without these longer-term analyses, it can be difficult to characterize change, let alone determine how to respond to it. Now that the effects of climate change are becoming clearer, it is time to focus on utilizing empirical and mechanistic modelling to fill in data gaps and optimally enhance understanding of climate change impacts and how to mitigate them. Climate change projection scenarios can be used in modelling sources of disease-related microbes, and fate and transport processes in coastal waters, to assess the effects of climate change on human health [141]. Modelling and QMRA can aid in understanding the risk level of microbial exposure in a changing environment [142]. Standardized surveillance and monitoring are still necessary to protect the health and safety of beachgoers on a daily basis. These data can also be used to supplement existing research, better inform, and drive models and shed light on climate change implications and management into the future.

Implementation of mitigation actions such as beach maintenance, informing beach users of risks associated with waterborne illness, and land use management will play a key role in protecting human health at beaches in the future.

## 3. Conclusions

Understanding the most critical climatic factors that influence changes in the microbiota of beach sands and recreational water, will aid in recommending best management practices to minimize the exposure risk and illness rates of the millions of individuals, who visit beaches to recreate each year. Some of these visitors may be immunocompromised at some level, which generates higher susceptibility to opportunistic microbes. If this is currently a reality, in a future reshaped by climate change, this type of knowledge may be much more relevant. Planning the future of recreational water use should consider sand and water, as well as the influences of climate changes in their microbiota.

## Figures and Tables

**Table 1:** Global mean sea level rise projections for 2100, under varying emissions scenarios [2].

RCP Emissions Scenario	Range of Projected Sea Level Rise (m)
RCP2.6	0.26–0.55
RCP4.5	0.32–0.63
RCP6.0	0.33–0.63
RCP8.5	0.45–0.82

**Table 2:** Examples of how predicted variability of environmental/climatic factors may affect pathogenic microbial exposure burdens and human health risks at the nearshore environment.

Hydrometeorological Variable	Projected Change in Variable	Impacts on Beach Microbes and Public Health	References
Air Temperature	Increased Air Temperature	Direct Effects:	[26,27,28]
		• Urbanization and development near water • Increased beach usage during very high temperatures	
		Indirect Effects:	[29,30]
		• Increased loading of microbes from additional human and animal shedding	
Water Temperature	Increased Water Temperature	Direct Effects:	[5,6,7,31,32,33,34,35,36,37]
		• Enhanced die-off of some taxa and shifts in microbial communities toward favouring more thermotolerant taxa	
		• Range shifts and expansions due to habitat suitability changes	[36,38,39,40,41,42,43,44]
		• Increases in harmful algal blooms occurrence and persistence (dinoflagellates and cyanobacteria) that may produce toxins and also provide refugia to other microbes	[19,22,31,32,45]
		Indirect Effects:	[46]
		• Changes to density, plume buoyancy, persistence and transport of microbes in the water column	
Precipitation	Increased Frequency and Intensity of Storm Events	Direct Effects:	[47,48,49,50,51]
		• Nearshore inundation causing the deposition of microbes into the sand	
		• Coastal erosion transferring sand-entrained microbes to the water	[52,53,54]
		• Stormwater runoff, introducing upstream microbes to the nearshore zone	[47,55,56]
		Indirect Effects:	[20,49,57]
		• Increased dissolved organic matter that changes solar inactivation dynamics and persistence of microbes in the water	
		• Infrastructure breakdowns leading to point sources of contamination at beaches (e.g., CSOs)	[50,58]
	Increased Drought Conditions	Direct Effects:	[59,60]
		• Accumulation of microbes due to lack of wash-out conditions	
		• Shifts in microbial communities to favour drought-resistant taxa	[15,61]
Sea Level	Sea Level Rise	Direct Effects:	[62,63]
		• Microbial exchange occurring further inland, potentially nearer to urban areas and with different microbial communities	
		Indirect Effects:	[64]
		• Flooding of gently-sloping terrain strands water and microbes onshore	
		• Microbial accumulation in sand biofilms exposed to tidal dynamics	[12,65]
Waves	Increased Wave Activity	Direct Effects:	[54,66,67]
		• Increased distribution of microbes over the beach face	
		• Tidal re-wetting may allow increased persistence in the sand	[54,68]

## Data Availability

Not applicable.
